# Glutathione Transferase P1-1 an Enzyme Useful in Biomedicine and as Biomarker in Clinical Practice and in Environmental Pollution

**DOI:** 10.3390/nu11081741

**Published:** 2019-07-27

**Authors:** Alessio Bocedi, Annalisa Noce, Giulia Marrone, Gianluca Noce, Giada Cattani, Giorgia Gambardella, Manuela Di Lauro, Nicola Di Daniele, Giorgio Ricci

**Affiliations:** 1 Department of Chemical Sciences and Technologies, University of Rome Tor Vergata, Via della Ricerca Scientifica 1, 00133 Rome, Italy; 2 UOC of Internal Medicine-Center of Hypertension and Nephrology, Department of Systems Medicine, University of Rome Tor Vergata, Via Montpellier 1, 00133 Rome, Italy; 3 PhD School of Applied Medical-Surgical Sciences, University of Rome Tor Vergata, Via Montpellier 1, 00133 Rome, Italy; 4 Section of Legal Medicine, Social Security and Forensic Toxicology, Department of Biomedicine and Prevention, University of Rome Tor Vergata, Via Montpellier 1, 00133 Rome, Italy

**Keywords:** glutathione, glutathione transferase, biomarker, cancer, neurodegenerative disease, liver disease, hemodialysis, chronic kidney disease, kidney transplantation, environmental pollution

## Abstract

Glutathione transferase P1-1 (GSTP1-1) is expressed in some human tissues and is abundant in mammalian erythrocytes (here termed e-GST). This enzyme is able to detoxify the cell from endogenous and exogenous toxic compounds by using glutathione (GSH) or by acting as a ligandin. This review collects studies that propose GSTP1-1 as a useful biomarker in different fields of application. The most relevant studies are focused on GSTP1-1 as a biosensor to detect blood toxicity in patients affected by kidney diseases. In fact, this detoxifying enzyme is over-expressed in erythrocytes when unusual amounts of toxins are present in the body. Here we review articles concerning the level of GST in chronic kidney disease patients, in maintenance hemodialysis patients and to assess dialysis adequacy. GST is also over-expressed in autoimmune disease like scleroderma, and in kidney transplant patients and it may be used to check the efficiency of transplanted kidneys. The involvement of GSTP in the oxidative stress and in other human pathologies like cancer, liver and neurodegenerative diseases, and psychiatric disorders is also reported. Promising applications of e-GST discussed in the present review are its use for monitoring human subjects living in polluted areas and mammals for veterinary purpose.

## 1. Introduction

Glutathione transferases (GSTs) represent a superfamily of multifunctional proteins expressed in almost all eukaryotic and prokaryotic cells, able to detoxify against endogenous and exogenous toxic compounds [1,2]. In mammalian organisms, they are grouped into three major families: cytosolic GSTs, mitochondrial GSTs, and microsomal GSTs [1]. Many different gene-independent classes represent the cytosolic GSTs; each group of GST isoenzymes presents similar sequences and structural properties. For example, in humans and mammals, seven classes are present i.e., Alpha, Mu, Pi, Theta, Omega, Sigma, and Zeta. While the Alpha class collects A1-1, A2-2, A3-3, A4-4 isoenzymes, the Pi class only contains one enzyme, the GSTP1-1 [2].

**Figure 1:**
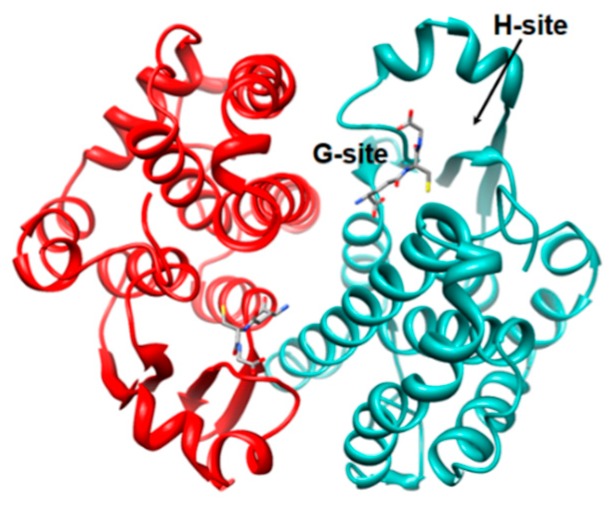
Structure of glutathione transferase P1-1 (GSTP1-1). GSTP1-1 (also referred: erythrocyte glutathione transferase) (PDB id: 6gss) [5]. The two monomers are in light-sea-green and red ribbons. Glutathione molecule is reported in ball-and-stick according to atom type. The G- and H-site are also shown only in one monomer.

These enzymes were discovered about sixty years ago [3]. Since then many studies defined structural and catalytic properties of various isoenzymes. All cytosolic GSTs are dimeric proteins that display similar tridimensional structures despite low sequence identity. Each monomer contains a binding site for glutathione (GSH) (G-site) and a second binding site for hydrophobic toxic compounds (H-site) (Figure 1) [4].

**Figure 2:**
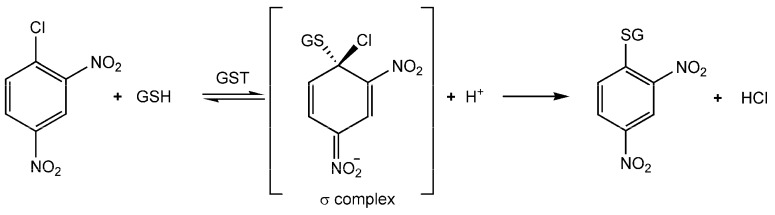
The conjugation of glutathione (GSH) to 1-chloro-2,4-dinitrobenzene (CDNB) catalyzed by GSTs. The formation of the product can be followed spectrophotometrically at 340 nm [7] where the product absorbs (ϵ_340_ = 9.6 mM^−1^ cm^−1^).

Three distinct GST subfamilies can be described on the basis of different protein residues able to activate GSH forcing its deprotonation: Cys-, Ser- and Tyr-based GSTs (Figure 2) [6].

**Figure 3:**
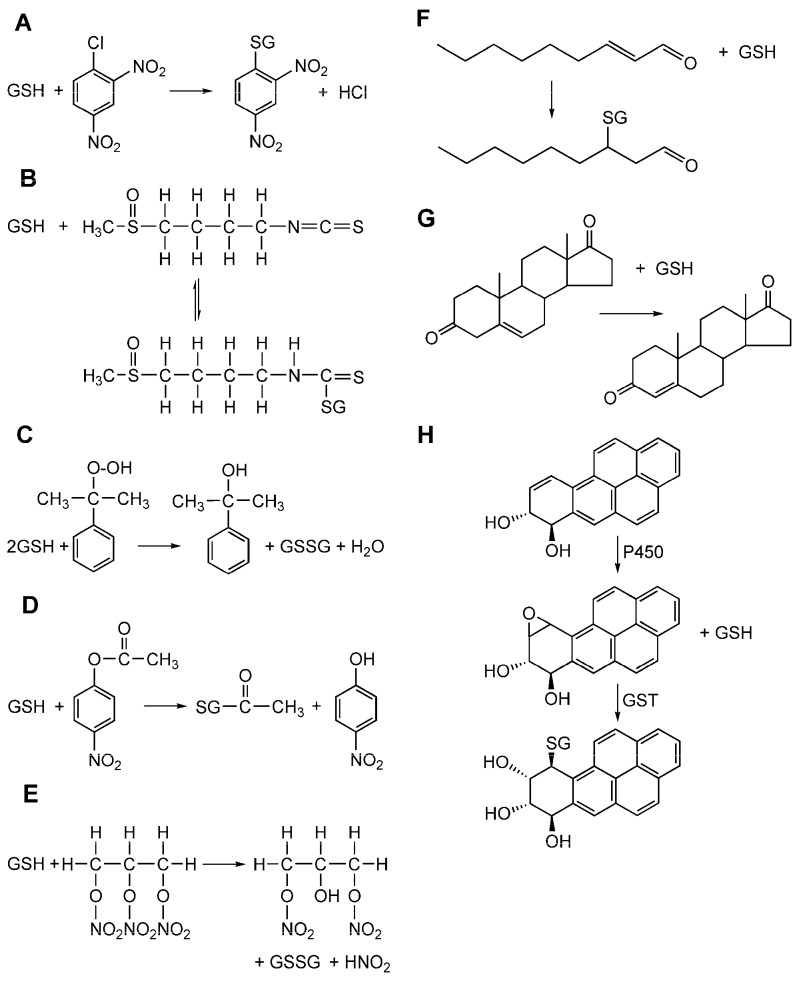
Examples of reactions catalyzed by GSTs toward glutathione (GSH) conjugation with different electrophilic substrates. (**A**) 1-chloro-2,4-dinitrobenzene (CDNB) is a GST substrate and represents an aromatic substitution reaction with glutathione; (**B**) sulforaphane, (**C**) glutathione peroxidase activity toward cumene hydroperoxide [8]. (**D**) 4-nitrophenyl acetate converted in alcohol, (**E**) trinitroglycerin. (**F**) *trans*-2-nonenal conjugated to glutathione by a Michael addition reaction [9]. (**G**) The double-bond isomerization of Δ^5^-androstene-3,17-dione into Δ^4^-androstene-3,17-dione, a precursor of testosterone [10]. (**H**) Reaction of detoxification from the polycyclic aromatic hydrocarbon 3,4-benzopyrene.

These enzymes, in fact, catalyze the nucleophilic attack of GSH to the electrophilic center of many toxic compounds with very different chemical structures (Figure 3).

**Figure 4:**
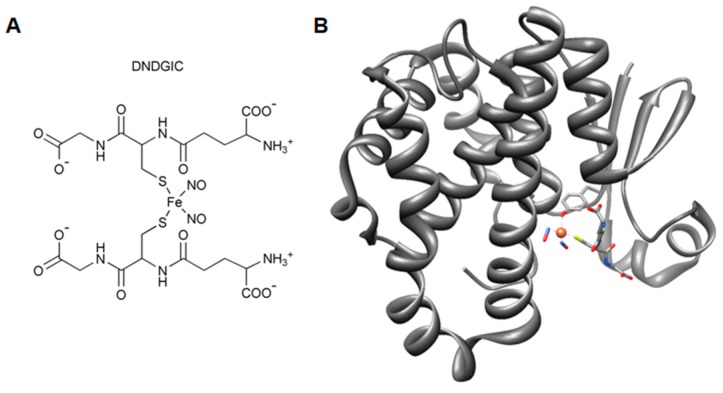
The interaction of the dinitrosyl-diglutathionyl iron complex (DNDGIC) with GSTP1-1. (**A**) chemical structure of DNDGIC. (**B**) three-dimensional structure of the dinitrosyl-glutathionyl iron complex (*ball-and-stick)* bound to a monomer of GSTP1-1 (*dim-grey* ribbons) (one GSH is replaced by a Tyr residue, which completes the coordination shell of the iron ion with its oxydril group) (PDB id: 1zgn) [12].

Some specific isoenzymes also display an additional selenium-independent peroxidase catalytic activity [11]. These enzymes may act as ligandins by binding and inactivating a variety of toxic compounds and peptides [4]. GSTs are also involved in the detoxification of a natural nitric oxide (NO) derivative, the dinitrosyl-diglutathionyl-iron complex (DNDGIC), a toxic compound which is formed in the cell in case of NO insults which becomes harmful when bound to GST (Figure 4) [6].

**Figure 5:**
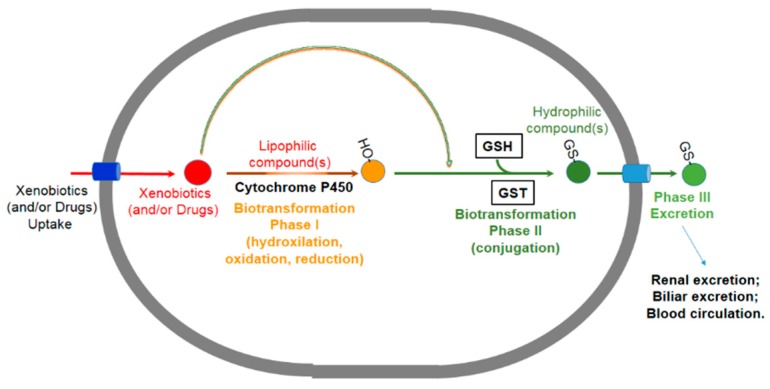
General biotransformation pathway of xenobiotics. Toxic compounds (e.g., endogenous, exogenous and drugs) inside the cell according to their chemical properties are taken over by the enzymes of different phases detoxification pathway. Lipophilic compounds are bio-transformed by Phase I enzymes (e.g., Cytochrome P450 family), more polar compounds are bio-transformed in Phase II reactions catalyzed by a second pool of enzymes (e.g., glutathione transferases). The final conjugated and more hydrophilic compounds will be transported out the cell by membrane channels, transporters and pumps (Phase III). Moreover, compounds with a polar or hydrophilic chemical nature may enter in Phase II or III respectively.

By considering that GSTs present in the cytosol of the mammalian cells account for about 5%–8% of all soluble proteins, they represent the most prominent defense line (Phase II) able to biotransform xenobiotics via enzymatic activity or to sweep dangerous toxins by binding them and promoting their extrusion from the cell (Figure 5) [13].

Interestingly, GSTs reach a 0.5–0.8 mM concentration in the cell so it works in vivo under the unusual conditions of [xenobiotic] << [GST]. As GST lowers the p*K*_a_ of GSH bound to the active site, it increases the concentration of deprotonated GSH in the cytosol by about five times thus accelerating its conjugation with toxins even if they are not typical substrates of this enzyme [13]. This catalysis becomes more evident in the case of cell acidification and GSH depletion [13]. The peculiar enzymatic conjugation of GSH to these toxic compounds is possible assuming a simple bimolecular collision between enzyme and substrate [13].

### 1.1. The Erythrocyte GSTP1-1 (e-GST)

The GSTP1-1 is present in many mammalian tissues including brain, heart, lung, testis, skin kidney, and pancreas. GSTP1-1 is also the most abundant intra-erythrocyte isoenzyme representing 95% of the entire GST pool [14]. Its x-ray structure was solved in our laboratory in collaboration with Parker and coworkers (Figure 1). Our group also studied its catalytic mechanism and defined many interesting structural and functional properties. This dimeric protein is composed of two identical subunits of about 23 kDa. Each subunit can be divided into two domains. The amino-terminal Domain I contains the binding site for GSH (G-site), and the carboxy-terminal Domain II is able to bind many different toxic compounds in a hydrophobic cavity (H-site). This enzyme also displays four cysteines, which do not form disulfide bridges. It follows a Michaelian behavior with a rapid equilibrium random sequential Bi-Bi mechanism [15]. Therefore, for many years this enzyme was considered a dimer with two structurally and kinetically independent G-sites. However, the replacement of Cys47 with alanine or serine decreased the affinity for GSH and triggered positive cooperativity for the binding of GSH [16]. This finding indicated a structural communication between subunits caused by the lack of a particular electrostatic bond between Cys47 and the protonated amino group of Lys54. The importance of Cys47 and its particular properties were also explored by means of simulated electrostatic potential measurements, which gave an unusual and very low p*K*_a_ of 3.5 [17].

The reactivity of this residue has been also used to probe the flexibility of helix-2, whose motions modulate both the affinity of G-site for GSH and the homotropic behavior of GSH in the mutated enzyme. Another residue, Tyr108, has been found to have a multifunctional action in the catalytic mechanism, depending on the nature of the electrophilic co-substrate [18]. A few other studies have been made to define the structure of GSH when bound to the G-site [19], and the crystal structure of GSTP1-1 in complex with various inhibitors [5]. A very interesting property of this enzyme is its kinetic and binding behavior at different temperatures. In fact, above 35 °C the binding of GSH to GSTP1-1 displays positive cooperativity, whereas negative cooperativity occurs below 25 °C. This mechanism minimizes changes of GSH affinity for the G-site because of temperature fluctuations [20]. This is an advantage for epithelial cells, rich in GSTP1-1 and exposed to temperature changes.

Other studies confirmed latent cooperativity in GSTP1-1 disclosed by the mutation of Gly41 and Gly50 [21]. More recent investigations discovered the involvement of GSTP1-1 in the storage and detoxification of NO. In fact, it was found that both S-nitrosoglutathione and the dinitrosy-diglutathionyl-iron complex, two well-known NO carriers, may bind and interact with GSTP1-1 [22]. In particular, the free DNDGIC is a toxic compound because it irreversibly inactivates glutathione reductase (GR) [23]. This complex binds with extraordinary affinity to the G-site (*K*_d_ = 10^−9^ M) and when bound to the G-site it becomes fully harmful. However, by means of negative cooperativity, when one subunit of the enzyme has bound DNDGIC, the other free subunit becomes unable to bind a second molecule [24]. This mechanism preserves GSTP1-1 from complete inactivation when it is involved in the DNDGIC detoxification, maintaining its classical conjugating activity even when an excess of NO is produced in the cell. This particular self-preservation has been also found also in other GST isoenzymes like the Alpha and Mu GSTs but not in bacterial GSTs, suggesting that this property has been acquired only in the more recently evolved organisms [25].

The property of GSTP1-1 to act as a ligandin can be extended in a certain way to the protein-protein interactions where this enzyme is involved in controlling signaling pathways and transcriptional responses of cells. The apoptotic signaling of Jun-kinase [26] and Bax [27] is under the influence of this interaction. GSTs also modulate calcium channels, decreasing the apoptotic mobilization of calcium ions [28]. Interactions of GSTP in the apoptosis include tumor necrosis factor-α (TNF-α), TNF-receptor factor 2 (TRAF2) and the apoptosis signal-regulating kinase 1 [29]. The activity of Peroxiredoxin-6 is also controlled in a redox-dependent manner by the interaction with GSTP, and evidence has been obtained on the existence of GSTP-dependent feedback of Nrf2 transcription factor activity [30]. GSTP1-1 is not only found inside the cell involved in the detoxification mechanisms and/or signal transduction pathways but it is also present in human fluids like saliva. In fact, GSTP1-1 represents the most abundant salivary GST isoenzyme, but it is present as an inactive oxidized form with two of its four cysteines linked as an intramolecular disulfide. The salivary hypothiocyanite is the main responsible for its inactivation [31]. Saliva remains the only biological compartment where GSTP1-1 has been recovered as an inactive oxidized protein.

### 1.2. GSTP1-1 in Blood

Serum only contains traces of GSTP1-1 (and other GSTs isoenzymes) [32]. Conversely, erythrocytes contain detectable amounts of GSTP1-1 (defined e-GST). Its normal concentration in humans corresponds to around 6 U/g Hb.

This enzyme appears to be inducible, i.e., its expression is modulated by levels of circulating toxins, and therefore, it represents a possible useful biomarker to verify the blood toxicity in all diseases associated with depurative organ dysfunction such as liver and kidneys [33]. Probably, this hyper-activity represents a defense response to the systemic toxicity in the uremic condition [34]. Interestingly, e-GST appears as a log-term biomarker as its concentration is determined in the early step of the erythropoiesis and its level does not change during the life of the erythrocyte.

## 2. Methods

The literature search conducted online databases (PubMed, Scopus, Web of Science) covered the following conditions: e-GST and/or GSTP1-1 as an enzyme, GSH in the detoxifying process and oxidative stress with a preference implication for the GSTP1-1. Furthermore, chronic kidney disease, kidney transplant, liver diseases, neurodegenerative diseases, cancer, environmental pollution, veterinary field, psychiatric disorders, as applications for the e-GST.

Graphics and histograms were obtained by GraphPad Prism (La Jolla, CA, USA). Three-dimensional structures of glutathione Transferases were drawn by the means of UCSF Chimera software v1.6 [35]. Chemical structures were designed by the software ChemDraw Ultra v8 (PerkinElmer Informatics, Cambridge, MA, USA).

## 3. Usefulness of GSTP1-1 Enzymatic Activity in Some Pathological Conditions

### 3.1. Over-Expression of e-GST in Chronic Kidney Disease

Several studies have shown an over-expression of e-GST in various diseases, including chronic kidney disease (CKD). The first study to monitor e-GST activity in nephropathic patients was conducted by Carmagnol et al. [33]. The authors showed that in neonates with hyperbilirubinemia and in hemodialysis (HD) patients (aged 7 to 20 years), a significant increase in e-GST activity was observed compared to age-matched healthy control subjects. Mimic-Oka et al. [34] confirmed this finding pointing out increased GST and GSH levels in red blood cell (RBC) and leukocytes of CKD patients either in pre-dialysis under conservative therapy and in hemodialysis.

Subsequently, Galli et al. [36] highlighted that enzymatic expression could be a useful biomarker to check the uremic toxicity status in CKD patients, hypothesizing that it could also be used for the evaluation of dialysis efficiency. The authors demonstrated that the enhancement of e-GST activity in uremic patients is a consequence of increased expression, rather than a kinetic modulation of the enzyme protein. In this observational study conducted on 118 patients, e-GST expression was higher in dialysis patients compared to the general population. The study also suggested that e-GST overexpression cannot be considered a surrogate marker of oxidative stress (OS) because it is not influenced by vitamin E supplementation. In the same manner, even the response to erythropoietin therapy apparently did not influence e-GST levels and preliminary data of this study suggested that high-molecular-weight or protein-bound toxins could play a key role in the e-GST overexpression. In the same study, only a few nephropathic subjects in pre-dialysis were examined and these patients presented a lower prevalence of e-GST overexpression when compared to HD patients (20% vs. 72%).

**Figure 6:**
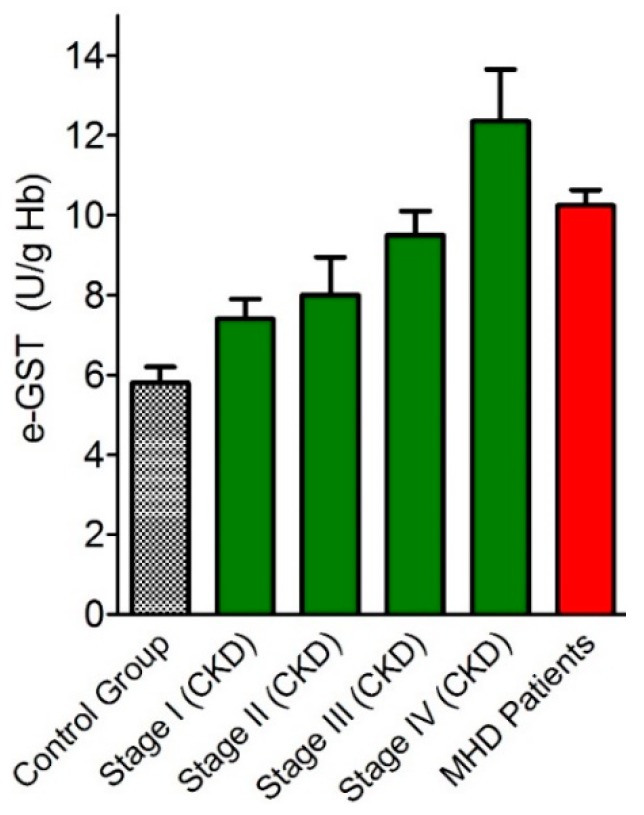
Erythrocyte glutathione transferase (e-GST) levels in healthy volunteers, chronic kidney disease patients and hemodialysis patients. Control group (healthy subjects), chronic kidney disease (CKD) stages I–IV (green bars); maintenance hemodialysis (MHD) patients (red bar) (modified from Reference [38]).

The data has been confirmed and further explained by a subsequent study, which analyzed the correlation of the degree of CKD, staging according to theNational Kidney Foundation Kidney - Disease Outcomes Quality Initiative (NFK K-DOQI) guidelines [37] with e-GST activity (Figure 6).

**Figure 7:**
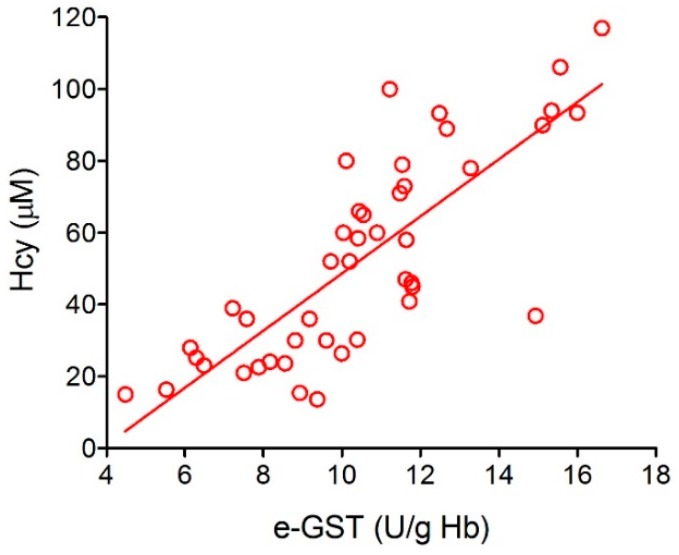
Linear correlation between homocysteine and erythrocyte glutathione transferase activity. Homocysteine (Hcy) is reported in μM and e-GST activity in U/g Hb (modified from Reference [38]).

The study was conducted on 72 CKD patients under conservative therapy. The results showed that the enzyme activity was higher with increasing disease severity and inversely correlated to the glomerular filtration rate (GFR). In the same study, e-GST was also assayed in 62 chronic HD patients. e-GST was always high but, somewhat surprisingly, its levels were significantly lower in chronic HD patients than in those with the IV stage of CKD (Figure 6). These findings can be easily explained by considering that the dialysis procedure is able to remove toxic compounds that accumulate during end-stage-renal disease (ESRD). Interestingly, e-GST activity was not related to the acute and chronic inflammation indices, nor to the nutritional status of the subjects. Conversely, direct correlation has been observed between the plasma values of homocysteine (Hcy) and the e-GST activity [38,39] (Figure 7).

Increased Hcy values lead to a decrease in nitric oxide synthase (NOS) activity. In fact, the self-oxidation of the sulfur amino acids of the Hcy leads to the formation of S-nitroso-homocysteine, which in turn inhibits the enzymatic activity of the NOS. For this reason, hyperhomocysteinemia was positively correlated with the increase in OS and endothelial damage [40].

**Figure 8:**
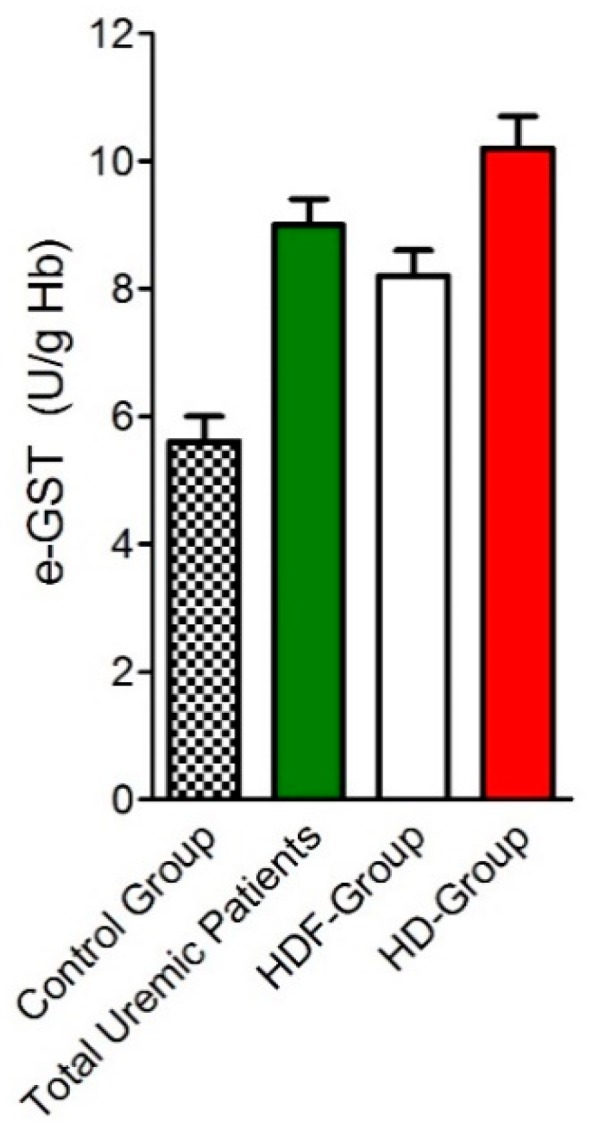
Erythrocyte glutathione transferase (e-GST) activity in uremic patients under two different dialysis techniques. e-GST activity in healthy subjects (control group), total uremic patients (green bar), patients in online hemodiafiltration (HDF-group) (white bar), and uremic patients in standard bicarbonate hemodialysis (HD-group) (red bar) (modified from Reference [41]).

A later study [41] verified the potential of e-GST as an alternative or complementary biomarker to the Kt/V_urea_ parameter in order to assess the dose and adequacy of dialysis treatment, comparing diffusive and convective dialysis techniques. It was underlined that e-GST activity does not evaluate the adequacy of a single dialytic treatment, as it occurs for Kt/V_urea_, but rather it represents a biomarker of dialytic adequacy for a number of dialytic sessions accomplished during a few weeks span. In this study, the increased e-GST activity in ESRD patients was confirmed in 103 HD patients compared to 82 healthy subjects (9.0 ± 0.4 vs. 5.6 ± 0.4 U/g Hb, respectively). Subdividing this population into two subgroups based on the type of dialytic procedure, 44 patients on diffusive techniques were compared with 59 patients on convective techniques. e-GST activity was significantly lower in convective than in diffusive subgroup (8.2 ± 0.4 vs. 10.0 ± 0.4 U/g Hb, respectively) (Figure 8).

Single-pool Kt/V_urea_ and total weekly Kt/V_urea_ were higher in convective group with respects to diffusive group (1.5 ± 0.1 vs. 1.3 ± 0.1, and 4.6 ± 0.1 vs. 3.9 ± 0.2), but no significant correlation was found between e-GST activity and Kt/V_urea_ data [41]. This data confirmed e-GST activity as a long-term marker of dialysis adequacy, even if further clinical studies conducted on a larger population will be necessary to definitively enforce such thesis.

A recent retrospective study [42] investigated plasma Hcy and blood thiol status of 98 HD patients. The study demonstrated that a daily (2 h) hemodialysis could lead to a better correction of the uremic retention solute than a standard (three times/week) HD. This correction effect of daily hemodialysis on hyperhomocysteinemia correlates with that on the detoxification enzyme e-GST and on plasma GSH [42].

The e-GST was also over-expressed in nephropathic patients with type 2 diabetes mellitus (T2DM). In fact, a recent study highlighted an increase in e-GST activity in nephropathic and non-nephropathic diabetic patients compared to the control group. Specifically, this increase was proportional to the stage of CKD. This study also confirmed the correlation between e-GST activity and the Hcy levels [43]. Therefore, e-GST could be considered an early biomarker of renal dysfunction in diabetic patients, as its overexpression could be present even in the absence of increased traditional renal damage markers (like albuminuria). In this context, possible correlations between traditional biomarkers, used for evaluation of glyco-metabolic control in T2DM, and e-GST activity were also examined. The results suggested that the overexpression of e-GST is related to the level of renal damage and not to diabetes itself [43,44,45].

This data differs from that observed in a previous study reporting no differences in e-GST activity between 68 T2DM and 32 non-diabetic patients [46].

### 3.2. Overexpression of e-GST in Kidney Transplanted Patients

Renal-transplantation represents the election treatment in uremic patients [47] as it improves the quality of life and reduces the risk of cardiovascular mortality and morbidity compared to chronic dialysis therapy [48]. In this category of patients is important to identify biomarkers of the intoxication status, of OS and of possible rejection in order to preserve the transplanted organ for as long as possible. In light of this, studies concerning the metabolism of glutathione and its related enzymes appear of great utility.

**Figure 9:**
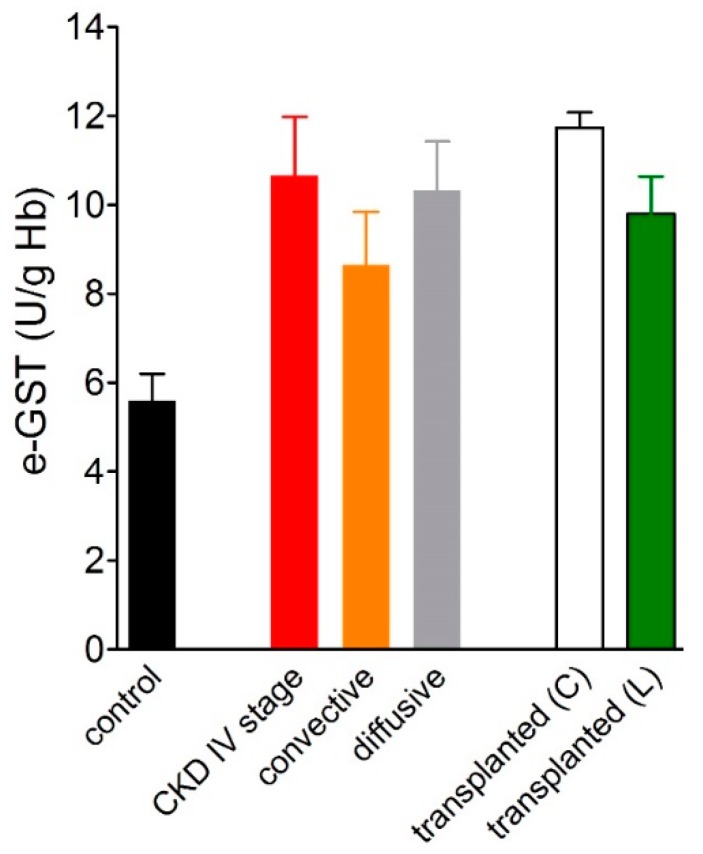
Erythrocyte glutathione transferase (e-GST) activity in transplant patients. e-GST activity of transplant patients from cadaver (C) (white bar) and living donors (L) (green bar) compared to healthy subjects (control) (black bar), CKD stage IV patients (red bar) and patients under two different dialysis techniques convective (orange bar) and diffusive (grey bar) (modified from Reference [49]).

In this context, a total of 169 kidney-transplanted patients after at least 3 months from transplant was examined: specifically 153 kidney-transplant patients from cadaver donors and 16 kidney-transplant patients from living donors [49]. Both groups had higher levels of e-GST activity when compared to the control group. In addition, the renal-transplanted patients from cadaver donors had significantly increased e-GST levels in comparison with patients receiving organs from living donors. The mean value of e-GST activity in the transplant patient, was comparable to that monitored in stage IV CKD patients (Figure 9).

These data suggest that during transplantation, the kidneys undergo an ischemia-reperfusion insult, which is observed in the course of the retrieval, losing part of their detoxifying capacity. This phenomenon appears more evident in transplanted kidneys from cadavers. In addition, OS and an inflammatory process are observed during renal ischemia-reperfusion: the lipid membranes undergo a process of peroxidation, while DNA and proteins suffer oxidative damage with consequent apoptosis and necrosis [50,51]. With the exception of steroids, no correlation was found between e-GST levels and immunosuppressive therapy and even with routine clinical and laboratory parameters. Furthermore, in one patient a large increase of e-GST value, about 180%, was observed just before acute rejection, supposing that it could become an early rejection biomarker [49]. This hypothesis should be confirmed by further clinical studies conducted on a higher number of patients.

### 3.3. GSTP1-1 in Neurodegenerative Diseases and Psychiatric Disorders

The central nervous system is particularly sensitive to OS because of the formation of reactive oxygen species (ROS) and the principal causes and effects of the high content of ROS are the alteration of the balance between pro- and anti-oxidant molecules and dysregulation of GSH homeostasis [52,53]. Neurons are active cells for their oxidative metabolism characterized by an equilibrium between supply and consumption of both glucose and oxygen, for such reason a crucial role for OS in the pathogenesis of neurodegenerative diseases was reported [54].

Neurological disorders are a large variety of pathologies including Alzheimer’s, Parkinson’s, epilepsy, and amyotrophic lateral sclerosis. In all these pathologies, *GSTP1* polymorphisms (Table 1) showed altered levels in term of decrease or increase [55].

Parkinson’s disease (PD) is a neurodegenerative disorder in which movement alterations and non-motor symptoms are present. In this pathological condition, a reduction in GSH levels may be involved in the onset of the disease [54], while GSTP1-1 levels increased in patients at advanced stages of the PD [56,57]. Moreover, *GSTP1-1* polymorphisms are associated with an increased risk of PD, following cigarette smoke [58], and pesticide exposure [59].

Alzheimer’s disease (AD) is a chronic neurodegenerative pathology, characterized by the accumulation of protein aggregates and fibrils in the brain. Recent studies suggest that GSTP1-1 is involved in cyclin-dependent kinase-5 regulation by the modulation of its expression in AD patients and therefore prevents neurodegeneration [60]. The presence of the allelic variant of GSTP1-1 (GSTP1-1 * C) may affect cognitive functions in certain AD patients and may be responsible for an increased susceptibility for late onset AD [61]. An important risk factor for AD may be the V allele of *GSTP1* mainly in the presence of *apoE 4* allele [62].

Epilepsy was defined as a cerebral disorder characterized by an enduring predisposition to generate epileptic seizures, and by the neurobiological, cognitive, psychological and social consequences [63]. The definition of epilepsy has been changed recently for more practical clinical use [63]. An evidence for the resistance to antiepilectic drugs derived from a correlation between increased level of GSTP1-1 in the brain and medical intractability of epilepsy. GSTP1-1 could be responsible for this condition of resistance in epileptic patients [64]. GSTs catalyze the conjugation of metabolites to GSH, favoring the removal of epoxide metabolites that are generated during the metabolism of antiepileptic drugs [65]. High levels of GSTP1-1 expression have been observed in endothelial and astrocytic cells in cases of intractable epilepsy, which would seem to be associated with resistance to antiepileptic drug treatment [65].

Furthermore, *GSTP1* polymorphisms and GSTP1-1 variants are involved in amyotrophic lateral sclerosis (ALS). ALS is an idiopathic, fatal neurodegenerative disease of the human motor system in which the pathophysiological mechanisms underlying the development of ALS seem multifactorial with a complex interaction between genetic and molecular pathways [66]. OS may cause ALS onset with the co-presence of heavy metal that trigger the increase of cellular ROS. Lead exposure and ALS risk may correlate with the expression of the GSTP1-1 (variant Ile105Val). This GSTP1 variant increased the effect of lead on the population of subjects examined. The association between blood lead levels and ALS was increased among GSTP1 variant carriers in fact differences in the phenotypic expression of GSTP1 in polymorphic variants may alter the clearance rate of lead-induced oxidative stressors and thereby influence a lead-ALS association [67]. Another study reported that mRNA levels for GSTP were significantly down-regulated in the spinal cord, motor cortex, and the sensory cortex of ALS patients [68].

Several studies have linked OS increase and the onset of schizophrenia [69,70]. Schizophrenia is a neurobiological disorder characterized by neurocognitive dysfunctions, it typically manifests as positive (for example hallucinations) and/or negative symptoms (cognitive dysfunction, decreased motivation) [71,72]. Two recent trials investigated GR and GST activities in both erythrocytes and platelets, in patients with schizophrenia. They concluded that the activity of glutathione-dependent enzymes is impaired in schizophrenia spectrum disorders and the decreased level of GR and GST contributes to a reduction in antioxidant defense. For this reason, the evaluation of GR and GST activities could be a novel potential biomarker for predicting treatment response in this population [73,74]. However, two studies did not find any association between *GSTP1* polymorphisms and schizophrenia, probably because *GSTP1* polymorphisms do not affect protein levels, but modulate GSTP1 affinity to its substrates. *GSTP1* polymorphisms do not confer susceptibility to schizophrenia [75,76].

Finally, the psychiatric disorder of autism (a neurodevelopmental syndrome) is defined by deficits in social reciprocity and communication, and by unusual restricted, repetitive behaviors. Autism is a heterogeneous condition with an intriguing medical debate about the cause that generates conditions associated to autism during childhood (from genetic predisposition to environmental exposition to toxin and many others) [77]. In this respect, studies were recently carried out about the correlation between autism spectrum disorders and detoxifying enzymes (like GST and in particular GSTP1). Interestingly, the role of *GSTP1*, *GST* theta 1, and *GST* mu 1 gene polymorphisms in susceptibility to autism spectrum disorders was investigated. In the population of children examined no significant associations was derived between autism spectrum disorders status and *GSTT1*, *GSTM1*, or *GSTP1* genotype. However, in children heterozygous for the *GSTP1* Ile105Val polymorphism, the odds of autism spectrum disorders were significantly higher in those with the null *GSTT1* genotype than those with the other genotypes [78].

### 3.4. e-GST Activity and Scleroderma

Scleroderma or systemic sclerosis (SSc) is an autoimmune disease, which induces connective tissue hardening. It determines vascular alterations, activation of the immune system and fibrosis of the skin and of internal organs [79]. In the pathogenesis of SSc, the exposure to toxins is proposed to play a pivotal role, since the endothelium damage is probably triggered by inflammatory cytokines, granzymes, ROS and vasculotropic viruses [80]. In fact, almost 70% of patients affected by SSc have a pulmonary dysfunction which represents the primary death cause in this population [81].

In this pathology, kidney damage is frequent, so the possible relationship between the degree of the disease and levels of e-GST activity was investigated. In fact, e-GST is overexpressed in all SSc patients (n = 102), reaching a mean value of 13 U/g Hb, more than two times higher than healthy subjects (5.8 U/g Hb). Enzyme levels in these patients correlated (r^2^ = 0.49, *p* < 0.0001) with the Medsger DSS [82] and DAI Valentini [83] indices that quantify the activity and severity of the disease. Surprisingly, e-GST levels of SSc patients were not influenced by the presence of kidney damage or by other defects of specific organs taken separately. Therefore e-GST hyper-expression in this condition appears to be linked with the exposure to putative toxins that cause the disease, rather than being caused by the autoimmune disease per se, by the damage of specific organs, or by other consequences of the disease that may also include OS [84].

The autoimmune diseases are not only limited to scleroderma but in medical science, more than one-hundred autoimmune diseases are classified. These disorders usually have a clear genetic component and evidence of activation of the innate immune system. The rates of autoimmune disorders are increasing in industrialized countries and greater attention is direct to improve diagnostic procedures and therapeutic interventions [85]. Only one study is based on the enzymatic level of e-GST in scleroderma [84]. The other studies reported in the literature focused on *GSTP1* polymorphisms (see Section 3.6 and Table 1) in pathologies like systemic lupus erythematosus [86], or are meta-analysis suggesting that the *GSTP1* polymorphisms are not associated with the risk of rheumatoid arthritis [87]. Another study confirmed the lack of association between *GSTP1* polymorphisms and multiple sclerosis [88]. However, further studies are required for a better comprehension of the environmental factors implicated and the roles played by *GSTP1* polymorphisms in the pathogenesis of autoimmune diseases.

### 3.5. Role of e-GST in Oxidative Stress

All subjects are chronically exposed to endogenous and exogenous oxidants species [89,90]. GST enzymes and other intracellular “redox buffers” provide protection representing an antioxidant network [91]. Compounds like ROS and OS are able to cause DNA, protein and lipid damage with an epidemic onset of chronic non-communicable diseases [92,93].

Various experimental studies have investigated the mechanism of action of various endogenous systems, including e-GST, which can promote defenses against OS.

A randomized controlled trial conducted in 2016 by Gouda et al. [94], investigated the activity of e-GST after 3 weeks intake of natural antioxidants, derived from plants polyphenols. The authors showed that e-GST activity increased significantly after consumption of plant polyphenols (derived from pomegranate juice) associated with fermented sour soya. These data suggest that a diet supplemented with a high content of antioxidants favors the body’s natural defenses against oxygen free radicals [95].

In a previous study [96], the effects of a low-protein diet in nephropathic patients on e-GST levels was investigated. This study highlighted a decreasing trend in e-GST mean values, although not in a statistically significant manner and an improvement in renal function assessed through estimated-GFR (e-GFR). Therefore, even in nephropathic patients, correct dietetic-nutritional treatment can be a valid therapeutic support to counteract the progression of CKD and the increase of OS.

A review in 2014 by Salminen et al. examined the different physiopathological mechanisms that could lead to brain aging [89]. OS plays a decisive role in the decline of cognitive function and in the aging process [97,98]. Compared to other organs, the brain has some disadvantages related to the generation and detoxification of ROS. In fact, brain cells use about 20% of body oxygen, even though they represent only 2% of total body weight [99]. Therefore, in the brain, there is a very high concentration of ROS and has moderate activity of catalase (CAT), glutathione peroxidase and superoxide dismutase (SOD) compared to the liver and kidney [100]. Moreover, in the brain, there is a superoxide accumulation, which is able to interfere with DNA structure and with the mitochondrial electron transport chain [101]. In this context, the action of glutathione is fundamental for the elimination of peroxides and free radicals in the brain cells and in the protection against ROS [102,103,104].

### 3.6. GSTP1-1 in Cancer

GSTs are one of the primary causes of cancer treatment failure. The problem of drug resistance (e.g., chemotherapy) may be attributed to factors of different nature like inhibition of apoptosis pathways, expression of multidrug resistance-associated proteins, altered drug metabolism or uptake [105]. Chemotherapeutic-resistant tumor cell lines have been shown to overexpress GST isozymes. GSTP1-1 is abundantly expressed in some mammalian tissues associated with tumors. GSTP1-1 usually is highly expressed in proliferating cells than in the differentiated cells and this elevated expression is associated with the cancer progression and therapy resistance [106]. This overexpression leads to accelerated detoxification of drug substrates and thus an acquired resistance. Furthermore, the roles of GSTP1-1 are not only limited to the catalytic properties but also to regulate kinase-dependent proliferation pathways; in fact, the ligand-binding capacity results in the negative regulation of signaling pathways through sequestration of signaling kinases [105]. The condition of OS in the cell favors the dissociation of the complex between GSTP1-1 and Jun-kinase and the subsequent activation of the released Jun-kinase allowing the induction of apoptosis. In tumor cells, kinase pathways are dysregulated, and so the cells may attempt to compensate by enhancing expression of GSTP1-1 to control kinase activity. The formation of the complex (GSTP1-1: Jun-kinase) is an event that protects tumoral cells from apoptosis [107].

The parallel overexpression of GSTP1-1 and efflux pumps may confer resistance to the tumor cells against chemotherapeutic drugs like cisplatin in osteosarcoma [108]. Another category of compounds in cancer research is the inhibitors of GSTP1-1 [109]. The inhibitors enhance the effect of the anticancer drugs and they may be used in novel therapeutic applications. The ethacrynic acid (a strong diuretic drug) is conjugated to 2-amino-2-deoxy-D-glucose to reduce diuretic effects but maintaining the inhibitory capacity against GSTP1-1, the ethacrynic acid derivatives are molecules with promising anti-proliferative activities against cancer cells [110]. Examples of well-characterized inhibitors of GSTP1-1 are auranofin and the irreversible inhibitor ethacraplatin. An interesting class of inhibitors is represented by GSH analogues that are more specific for GSTs and less toxic for the cell. An example of a GSH analogue was obtained through the chemical modification of γ-L-glutamyl-L-cysteinylglycine (GSH) into γ-glutamyl-S-(benzyl)cysteinyl-phenylglycine diethyl ester (i.e., ezatiostat or TLK199) that is easily absorbed by the cell where its metabolites bind the G-site (the GSH binding site) of GSTP1-1 causing its inhibition [111]. Selected 7-nitro-2,1,3-benzoxadiazole derivatives have been characterized as very efficient inhibitors of GSTP1-1. In particular, 6-(7-nitro-2,1,3-benzoxidiazol-4-ylthio) hexanol (NBDHEX) is an efficient inhibitor able also to dissociate GSTP1-1 from its complex with Jun-kinase or TRAF-2. NBDHEX stimulates proapoptotic pathways with an anticancer capability also showing activity on cisplatin-resistant human osteosarcoma cells [108].

Chemotherapy, the most common therapeutic treatment for cancer, shows two main limitations due to dose-limiting toxicities of drugs and the development of drug resistance. Therefore, research studies have been focused on classes of natural products that can be used as potential anti-cancer agents. Botanical sources, phytochemical classes and chemical structures of these natural products together with their influence on GSTs induction in vitro and in animal models were studied [112]. In fact, a typical natural product, the piperlongumine isolated from *Piper* species is used in traditional medicine. Piperlongumine is hydrolyzed within the cell giving the active form and the latter interacts with GSH forming a complex that binds the active site of GSTP1-1 inhibiting the enzyme [113].

In addition to the inhibitors and chemotherapeutic drugs for GSTP1-1, there are the pro-drugs.

The pro-drugs specifically designed to interact with GSTP1-1 are divided into two groups: compounds that contain GSH or GSH-like structure, and molecules activated by the formation of GSH-conjugate intermediate via GSTP1-1 enzymatic activity. In the first group, the canfosfamide is a GSH analogue activated by GSTP1-1 and in the other one, doxorubicin derivatives are converted in the active parent drugs via sulfonamide cleavage by GSTP1-1 [114].

The studies focused on GSTP1-1 and its relationships with cancer biology were not limited to finding a way to inhibit the enzymatic activity or modulate the apoptotic pathway with the development of different compounds. The molecular biology of *GSTP1* gene expression, the transcript levels and the enzyme expression in different types of tumors is a new frontier of research.

The polymorphism of *GSTP1* could be considered as single nucleotide point mutations within exon 5, in which the most common are Ile105Val and Ala114Val. The mutated enzyme shows change at the substrate-binding site but without affecting the GSH-binding affinity (Table 1). These polymorphisms influence the enzyme activity consequently the drug detoxifying capacity altering the cellular DNA damage and indirectly enhancing the risk of cancer development [106,107]. Generally, Ile105Val polymorphism is associated with a higher susceptibility to a variety of malignancies but also the Ala114Val polymorphism contributes to cancer risk susceptibility as it appears in esophageal carcinoma.

Nevertheless, *GSTP1* expression varying through methylation state of the specific CpG islands is not recognized in other *GSTs* genes. Hyper-methylation of the promoter region has been reported in human prostatic carcinomas, but not in normal or benign tissues. Aberrant methylation in breast cancers and renal carcinomas has been observed. In all cases, methylation was associated with loss of GSTP1 expression [104]. High prevalence of *GSTP1* gene methylation has been found in the serum of gastric cancer patients. This methylation detected in serum, possibly caused by circulating nucleic acid released by gastric cancer cells, is correlated with gene methylation in gastric cancer tissues [115]. GSTP1 represents an ideal epigenetic biomarker and may be used as a liquid biopsy biomarker. Indeed, it could be detected with good results in circulating cell-free DNA and urinary DNA. This promising future clinical application may be of interest because methylation of *GSTP1* can be found in the early event of carcinogenesis representing a sort of early biomarker in different tumors [116].

Actually, the scientific literature about the *GSTP1* polymorphisms and cancer is extremely abundant, in this respect here we only report a few examples for a restricted number of common types of tumors.

The important class of blood tumors is the first in which polymorphisms are associated to poor prognosis and methylation state of *GSTP1* promoter. The Hodkin’s lymphoma was studied for the involvement of *GSTs* polymorphisms in term of susceptibility and progression and also for the prognosis [117]. Leukemia studies were focused on the association of Ile105Val polymorphism with chronic myeloid leukemia [118] and on genotypes of *GSTP1* Ile105Val substitution for both acute lymphocytic leukemia and acute myeloid leukemia patients. Notably, Val/Val might be considered as risk genotype for developing acute lymphocytic and acute myeloid leukemia associated with a poor prognosis [119]. The epigenetic control of *GSTP1* gene results relevant in cancer prevention and diagnosis. A correlation between promoter hyper-methylation of *GSTP1* and response to chemotherapy in diffuse large B-cell lymphoma proving that *GSTP1* gene methylation status could be an indicator of drug response and a prognosticator for this lymphoma [120]. In the specific case of multiple myeloma, no significant association was found between NAD(P)H:quinone oxidoreductase 1 Pro187Ser or GSTP1 Ile105Val polymorphisms and multiple myeloma risk and also *GSTP1* allelic variation may not influence susceptibility to this malignancy. Another study found no association between *GSTP1* Ile105Val or Ala114Val genotype and an increased risk of multiple myeloma but suggested that polymorphic variation in GSTP1 are significant predictors of outcome following treatment with chemotherapeutic agents and may be a step in the development of more individualized treatment regimens for myeloma based on host genetic factors [121].

Overexpression of GSTP1-1 is involved in poor prognosis in brain tumors including glioma and glioblastoma [65]. In brain tumors patients with anaplastic glioma who have *GST* genotypes encoding for a lower activity enzymes may confer a survival advantage respect patients who have higher activity genotypes [122]. A controversial conclusion in glioma emerges from another study in which the analysis did not find any association among GSTs and in particular the *GSTP1* polymorphisms (Ile105Val and Ala114Val) and tumor risk. These negative results support the evidence that *GST* genotypes may not be accurate predictors of tissue-specific GST expression as it occurs also for GSTP1-1 [123].

The role of *GSTP1* polymorphisms in solid tumor breast cancer is not well defined due to preliminary results deriving from two meta-analyses on a large number of women. A first study shows that *GSTP1* Ile105Val polymorphism may be associated with an elevated breast cancer risk in the Asian population [124]. Another study proposes that women who were homozygous for the variant *GSTP1* Ile105Val allele had a reduction in mortality risk [125]. The methylation state of *GSTP1* is also involved in breast cancer, in fact, the unmethylated state is a benign group while hyper-methylated *GSTP1* gene promoters represent a borderline/malignant tumor group of patients. GSTP1 expression can predict pathological response to chemotherapeutic treatments with 5- fluorouracil/epirubicin/cyclophosphamide in estrogen receptor-negative tumors but not in estrogen receptor-positive tumors [120].

The reproductive female apparatus is subject to a variety of tumors; GSTP1-1 is also studied in correlation with women patients affected by cervix and ovarian cancer. The expression levels of mRNA of the resistance genes, like GSTP1, were measured in cancer tissue specimens and compared with pathological data, to understand their role in primary drug resistance. The mRNA expression levels of GSTP1 in cervical cancer tissue specimens were higher with respect to the healthy cervical tissues. In conclusion, GSTP1 mRNA levels in the tumor tissues did not exhibit a significant association with the clinicopathological features of the patients but only mediating resistance of tumor cells to cisplatin [126]. The ovarian cancer studies were based usually existing upon patient controls and hospital-based study designs. Unfortunately, the studies carried out do not confirm an association between GSTP1 and epithelial ovarian cancer [127]. In general, no consistent association between any gene polymorphism and clinical outcome in gynecological cancers has been found across studies [128]. Endometrial carcinoma is the most common gynecologic cancer in developed countries; curiously a first study reported an association between *GSTP1* Ile105Val polymorphism and endometrial carcinoma. Whereas a statistically significant association was shown between *GSTP1* polymorphism and type I endometrioid carcinoma of endometrium, no significant association between *GSTP1* polymorphism and non-endometrioid type II cancer could be established [129].

The global burden of prostate cancer is substantial, ranking among the top five cancers for both incidence and mortality and globally, prostate cancer is the most commonly diagnosed cancer in men [130]. *GSTP1* gene expression and GSTP1-1 enzyme activity were studied in human prostate carcinoma cells and human prostate tissue specimens. The results suggested that *GSTP1* promoter methylation is higher in cancer tissue than in benign tissue from the same individual and reduced GSTP1 expression is observed in prostate cancer specimens compared to their benign counterparts. The loss of GSTP1 expression in human prostate cells increased their susceptibility to OS-induced DNA damage [131]. Detection of *GSTP1* methylation in all types of body fluids of prostate cancer patients represents a promising epigenetic biomarker, while the unmethylated promoter allowed to distinguish benign lesions from cancerous transformations [120].

The renal cell carcinoma and transitional cell carcinoma of the urinary bladder studies offered experimental data to determine if the GSTP1-1 may be considered a potential urinary marker [132]. GSTP1-1 is generally present in renal cell carcinoma; however, the level of expression has been reported to be increased, unchanged or decreased compared with normal kidney tissue. Generally, data supported that GSTP1-1 activity contributes to the intrinsic drug resistance in this tumor [132]. GSTP1-1 overexpression is characteristic of transitional cell carcinoma of the urinary bladder. Detectable levels of urinary GSTP1-1, deriving from desquamation of the tumor, have been addressed in only one study as a potential urinary marker [133]. Plasma GSTP1-1 could be considered as a marker of transitional cell carcinoma of the urinary bladder, in fact, elevated levels of GSTP1-1 were found in patients with tumors. However, the conclusion of the study was clear against the possible use of plasma GSTP1-1 as a marker of bladder cancer [134].

Colorectal cancer is the third most common form of cancer and the fourth most frequent cause of cancer deaths worldwide. The overall survival, GSTP1-1 expression, and *GSTP1* genetic polymorphism in stage C of colon cancer were investigated in patients after resection alone versus patients after resection treated by a 5-fluorouracil-based chemotherapy. Stage C colon cancer patients with high GSTP1-1 should be treated with 5-fluorouracil-based chemotherapy on the other hand patients with low intracellular concentrations of GSTP1-1 may not need to be treated. This study highlighted the possible predictive value of GSTP1-1 expression in regard to chemotherapy for stage C colon cancer [135]. Meta-analysis studies did not confirm previous observations about a role for *GSTP1* Ile105Val polymorphism in colorectal cancer susceptibility [136] and the capability of *GSTP1* Ile105Val polymorphism to confer any additional colorectal cancer risk [137].

Gastric cancer is a multifactorial disease involving genetic, epigenetic, and environmental factors, including diet, chronic atrophic gastritis, radiation exposure, and infection by *Helicobacter pylori* [138]. *GSTP1* polymorphism was significantly associated with gastric cancer suggesting that can be considered a risk factor associated with gastric carcinogenesis [139]. Moreover, the same *GSTP1* polymorphism associated with larger tumor size may contribute to cancer progression and aggressiveness [140]. Conversely, in a population examined for gastric cancer there was no relationship with polymorphisms in *GSTP1* [141] and for another study patients with gastric cancer who received 5-fluorouracil/oxaliplatin chemotherapy as a first-line treatment, those possessing the *GSTP1* 105Val variant allele showed a statistically significant benefit for both time for progression and overall survival [142].

Esophageal squamous cell carcinoma is a lethal malignancy and there are few useful markers for its diagnosis and treatment. In a recent study, there was a significant association between GSTP1 expression in resected tissue and biopsy samples in patients with esophageal squamous cell carcinoma without neoadjuvant chemotherapy. GSTP1 was related to malignant potential and may be a predictive marker of drug resistance in esophageal squamous cell carcinoma patients [143]. A previous study found a significant association between the variant *GSTP1* Ala114Val genotype and increased risk of recurrence and death. GSTP1-1, which is actively involved in the detoxification of cisplatin, has been implicated as a predictive marker of overall survival in cancer patients receiving cisplatin-based chemotherapy [144]. Finally, GSTP1 is a major GST isoform expressed in the human esophagus but a review on the argument stated that results of relations between *GSTP1* polymorphisms and esophageal cancer were inconsistent [145].

Pancreatic cancer is a multifactorial disease with metastasis-prone and therapy-resistant nature; the predominant expression of GSTP1-1 in pancreatic cells may explain why *GSTP1* polymorphisms exerted effects on risk and survival of pancreatic cancer. The first study to suggest a role of *GSTP1* polymorphisms in pancreatic pathogenesis concluded that genetic polymorphisms of *GSTP1* may be among the mechanisms that modify the risk of pancreatic cancer in older individuals and affect the survival of patients who receive 5-fluorouracil-based treatment [146]. Indeed, tumor expression of GSTP1 does not predict the safety or efficacy of platinum-based chemotherapy regimen Folfirinox (leucovorin, fluorouracil, irinotecan, and oxaliplatin) in patients with pancreatic cancer [147].

Lung cancer is the most morbid and mortal disease among tumors, recent studies demonstrated that only *GSTP1* Ala114Val polymorphism, but not *GSTP1* Ile105Val polymorphism or wild-type genotype, was associated with improved survival in non-small cell lung cancer patients. In addition, no significant association between *GSTP* polymorphisms response to first-line platinum-based chemotherapy was observed in the patients examined [148,149]. A meta-analysis indicates that the risk of lung cancer is not associated with the Ile105Val and Ala114Val polymorphisms in the *GSTP1* gene [150]. However, many studies were not in agreement about the *GSTP1* methylation frequency in cancerous tissue of non-small cell lung cancer patients respect adjacent benign tissue [116].

Skin cancer can be divided into melanoma and non-melanoma skin malignancies. Most skin cancers are non-melanomatous including basal cell carcinoma and squamous cell carcinoma. Melanoma only accounts for about 2% of malignant skin cancer but causes most deaths [151]. Moreover, environmental and genetic factors influence the development of disease. *GSTP1* Ile105Val polymorphism may have a genetic contribution to the development of malignant melanoma [152]. A previous study confirmed the association of Ile105Val polymorphism with malignant melanoma [153]. Induced skin tumors may be affected by exposition to toxic compounds like arsenic compounds mainly the inorganic trivalent form (arsenite). An analysis of skin cancer patients suggested that GSTs (including P1-1), ROS, related metabolic genes, and DNA repair genes together may play a role in arsenic-induced skin carcinogenesis [154].

Osteosarcoma is the leading cause of bone malignancy in adolescents. The etiology of osteosarcoma is not well understood; in fact, environmental (e.g., ionizing radiation) and genetic factors may contribute to the development of this cancer [155]. One of the most recent studies, in which Asian osteosarcoma patients were analyzed, suggested that the *GSTP1* gene polymorphism is associated with an increased risk of osteosarcoma, whereas the other *GSTs* gene polymorphisms may not influence the development of this cancer [156]. Another previous study found a significant association between the polymorphisms GSTs and osteosarcoma risk, but no evidence of association about *GSTP1* polymorphisms with prognosis in osteosarcoma [157]. Furthermore, a study suggested that genetic variation of *GSTP1* Ile105Val may be used as a prognostic factor to identify osteosarcoma patients who might benefit from chemotherapy [158].

In conclusion, all studies about GSTP1-1 and cancer could be divided into two groups. In the first one, the over-expression of GSTP1-1 enzyme in cancerous cells is a well-defined and understood phenomenon. Drug development is focused on GSTP1-1 enzymatic inhibition, new potential chemotherapeutic agents, and pro-drug molecules. The second group, exploring the molecular biology and *GSTP1* gene polymorphisms, comes to controversial conclusions about the tumor risk factor associated with *GSTP1* polymorphism due to the small sample size of the population examined in the majority of published studies and the absence of an associated statistical analysis. GSTP1-1 remains a promising biomarker but not already used in the clinical practice.

### 3.7. GSTP1-1 and Liver Disease

Although the GSTP1-1 enzyme is not expressed in human hepatocyte under physiological conditions, it may be present in some pathological conditions such as liver cirrhosis or hepatocellular carcinoma (HCC) [159].

As previously discussed, the first study that investigated the enhancement of e-GST activity in liver diseases was conducted by Carmagnol et al. [33]. This study pointed out that e-GST was higher in neonates presenting hyperbilirubinemia at birth compared to normal neonates.

Further studies have highlighted, in animal models, the relationship between the expression of GST and the presence of liver fibrosis. In human, liver fibrosis is frequently associated with HCC, but little is known about the involvement of the fibrosis in carcinogenesis.

Sakaida et al. [160] investigated the effect on liver fibrosis of the administration of a prolyl 4-hydroxylase inhibitor (HOE 077) to male Wistar rats fed with a diet deficient in L-defined amino acids (CDAA) choline. The HOE 077 administration reduced the hepatic content of hydroxyproline (a parameter that reflects the amount of collagen), the number of pseudolobules and made the fibrous septa thinner. Furthermore, it reduced the number, average, diameter and percentage of GSTP-positive lesions.

In a later study, the same group of researches [161] showed how the injection, for eight weeks, of pig serum in rats induced stellate activation resulting in hepatic fibrosis without obvious parenchymal cell damage. Moreover, a CDAA diet for six weeks caused pre-neoplastic lesions both in pretreated and non-pretreated rats with pig serum. These pre-neoplastic lesions were positive for the placental form of GST (GSTP).

Subsequently, these authors further deepened the studies [162] by detecting how TGF-β1 RNA expression is induced by treatment with pig serum and inhibits hepatocyte proliferation, but it does not prevent the development of pre-neoplastic lesions in a CDAA diet model.

Hepatic cancer is the second leading cause of cancer death [163]. A large number of studies investigated the possible correlation between the cancer onset and the increase in GSTP levels.

Dysregulation of the Kelch-like ECH-associated protein 1 (Keap1)-Nrf2 pathway has been showed in experimental and human tumors, suggesting its possible role in cancer development. In an experimental rat model, that induces diffuse fatty liver and steatohepatitis with fibrosis, Orrù et al. [164] investigated how the mutation/activation of Nrf2 is involved in early stages of hepatocarcinogenesis. Its inactivation leads to prevent the development of pre-neoplastic lesions, identified by increasing of GSTP.

Kin et al. [165] evaluated the effect of TNP-470 (an inhibitor of angiogenesis) on the progression of HCC in an experimental rat model. In CDAA diet rats, TNP-470 treatment caused a reduction of the size, the frequency and vascularity of HCC compared to untreated rats. However, TNP-470 treatment did not influence the histology of liver cirrhosis and liver function. The authors concluded that TNP-470 did not affect the proliferation and apoptosis in GSTP- positive precancerous lesions.

Yang B et al. [159] examined the correlation between HCC and the promoter methylation status of nine tumor suppressor genes (TSG) (such as SOCS-1, GSTP, APC, E-cadherin, retinoic acid receptor beta, p14, p15, p16, and p73). Around 53% of the HCC cases had three or more TSG promoters methylated, in particular, methylation of SOCS-1 (65%), GSTP (54%) and APC (53%) was significantly more frequent in HCC compared to the cirrhotic liver (*p* < 0.05).

Perra et al. [166] assessed that α-lipoic acid (α-LA), administered in a diet deficient in choline and methionine (CMD), promotes the growth of hepatic pre-neoplastic lesions in an animal model of hepatocarcinogenesis. Pre-neoplastic lesions were identified due to their positivity to GSTP. The administration of α-LA to rats fed CMD diet significantly increased the number of GSTP-positive lesions compared to rats treated only with the CMD diet, the average size of the positive areas to the GSTP and the percentage of GSTP-positive liver tissue. Moreover, the treatment of α-LA in combination with CMD diet caused fat accumulation, lipid peroxidation and hepatocyte death, greater expression of tumor necrosis factor-α, cytochrome 2E1, cyclooxygenase-2, chronic hepatocyte proliferation.

Lee et al. [167] found, in the rat liver, increased expression of EGF-R (transmembrane tyrosine kinase receptor) and reduced expression of receptor tyrosine-protein kinase ErbB4 during diethylnitrosamine (DEN)-induced hepatocarcinogenesis. The EGF-R expression is related to hepatocyte proliferation caused by DEN through the nodule’s formation and hepatocellular neoplasms. In this rat model, the authors used GSTP as a biomarker to evaluate the presence of neoplastic foci. In fact, in most HCC, GSTP was expressed focally.

A study conducted in 2010 by Chen Y et al. [168] in the Taiwanese population investigated the possible genetic polymorphisms that can increase the risk of developing HCC. This study concluded that AG and GG alleles of *GSTP1* gene polymorphism may increase the risk of developing HCC in the population aged < 57 years.

A recent meta-analysis highlighted that *GSTP1* hypermethylation induces the inactivation of the *GSTP1* gene, plays a pivotal role in hepatocarcinogenesis, and is associated with an enhancement risk of HCC [169].

Wang et al. [170] evaluated the prognostic value of single nucleotide polymorphisms in seven encoding genes of GSTs for HCC. Rs4147581 in *GSTP1* gene had a significant relationship with the survival of HCC patients (*p* = 0.006), while its mutant allele presented a significantly lower risk of death compared to homozygous wild-type. The authors highlighted the role of *GSTP1* rs4147581 polymorphism as a prognostic indicator of HCC.

## 4. Environmental and Endogenous Factors Affecting GST Levels in Healthy Subjects

Environmental pollution is one of the most serious global challenges that affects biodiversity, ecosystems, and human health worldwide. Most pollutants have various adverse health effects from early life; some of the most important harmful effects are perinatal disorders, infant mortality, respiratory disorders, allergy, malignancies, cardiovascular diseases, increase in OS, endothelial dysfunction, mental disorders, and various other harmful effects [171,172].

**Figure 10:**
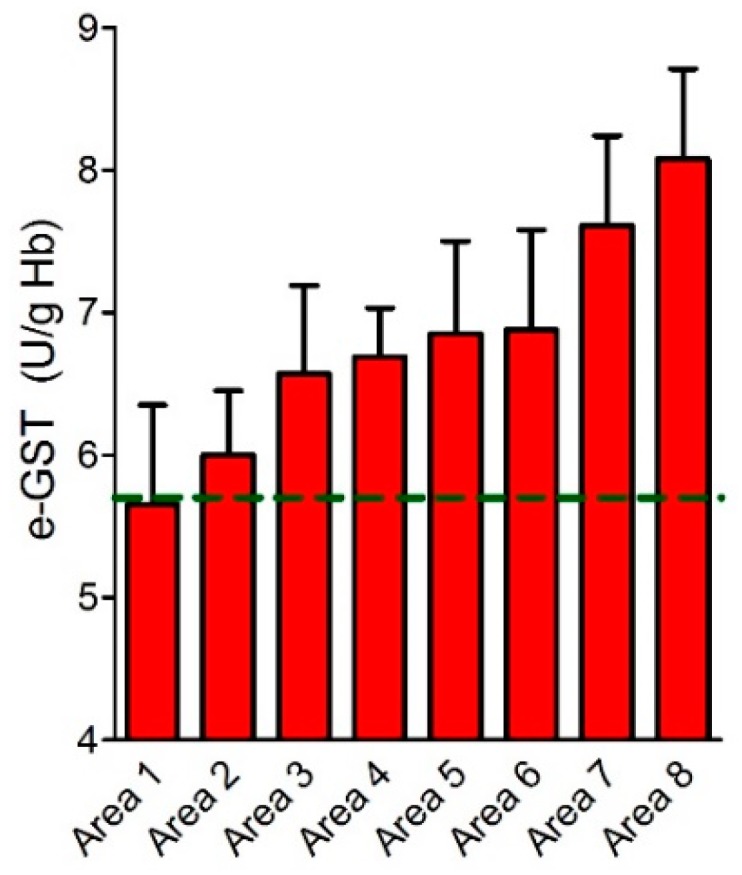
Erythrocyte glutathione transferase (e-GST) activity in the Sacco River valley. e-GST activity in the population of eight selected areas monitored (area 1–8, red bars) compared to the e-GST activity reference value for the Rome area (dotted green line) (modified from Reference [177]).

GSTs have been widely used as a biomarker of pollution response. Accordingly, careful studies of this kind of enzyme especially on fishes, have been of great importance due to ecological and toxicological reasons [173,174,175,176] e-GST is overexpressed, in healthy subjects living in polluted areas (Figure 10) [177].

e-GST can be used as a prognostic biomarker and monitoring tool of human conditions associated with increased exposure to endogenous toxins, xenobiotics and OS.

e-GST has been used as a biological marker of chemical exposure to industrial toxicants. Ansari and coworkers [178] showed that important industrial chemicals (propylene oxide, styrene oxide, ethylene dibromide and ethylene dichloride) inhibited GST from erythrocytes both in situ and in purified GST. This suggests that chemical exposure results in the reduced capability of e-GST to detoxify xenobiotics.

Singh and Awasthi [179] showed that 1.3 mM of the herbicide 2,4-dichlorophenoxyacetate suffices to inhibit 50% of purified GSTP1-1. The in vitro susceptibility of GST activity from human erythrocytes to industrially important electrophiles like acrolein, propylene-oxide, styrene oxide, ethylene dibromide and ethylene dichloride has been described by Ansari et al. [178].

Kilpikari and Savolainen [180] in 1984 reported decreased values of e-GST activity in workers exposed to hot rubber fumes.

A more recent study on about 500 healthy volunteers, living in eight distinct areas at or near the Sacco River valley, a region of the Frosinone district (Lazio, Rome, Italy) (Table 2) well known for its environmental pollution, proposed a role for e-GST as a biomarker of environmental pollution hazard.

Subjects from six different areas of that region showed 18%–44% increased levels of e-GST when compared to 400 volunteers living in the Rome hinterland, and the highest GST levels were observed in the areas of higher risk of pollution (Figure 10). Oxidation dependent changes of GST activity were not observed in the blood specimens of the exposed populations [177].

Primavera et al. [181] studied workers exposed to 1,3-butadiene, a well-known oxidizing compound also present as a contaminant in the air of a few industrial areas, and probably also to other chemicals. The results showed that occupational exposure to low doses of 1,3-butadiene and probably also to other chemicals may indeed induce OS and impair GST balance in the RBC of workers and, therefore, suggest that the measurement of GST activity and of the glutathionylated hemoglobin levels can be recommended as promising biomarkers in petrochemical workers.

Moreover, *GST* genes are involved in oxidative stress management and may modify the impact of indoor air pollution. Indoor air pollutants (e.g., tobacco, smoke, dust, and generated from cooking and heating) may contribute greatly to allergic disease pathogenesis in people who spent much of their time indoors [182]. The authors conclude in the systematic review that *GSTP1* Val genotypes are more susceptible to indoor air pollution exposure, having a higher risk of asthma and lung function deficits, although some findings are conflicting in terms of risk alleles and specific exposures [182].

### e-GST in Acute and Chronic Exposition to Contaminants: A Brief Comment

On the basis of the current literature, it appears that a chronic exposition to various contaminants may increase or decrease the activity of e-GST. When an increase occurs as in the case of the humans living in polluted areas or exposed to pesticides [183]; this is likely due to an increased synthesis of e-GST and to the absence of specific GST inhibitors in those contaminants. On the contrary, chronic exposition to specific toxins that may inhibit the enzymatic activity of GST may cause an underestimation of the e-GST which instead has been over-expressed in the erythrocyte. This has been likely demonstrated to occur for smokers [184].

In fact, e-GST activities of 3.03 ± 0.18 U/mg were found for the 46 smokers, and this value is significantly lower than what was found for the 41 non-smoker controls (3.98 ± 0.26 U/mg). This lower activity value in smokers is in accordance with a previous study [185]. However, elevated GSTP protein concentrations in smokers were found using an ELISA technique. This suggests that an extra GST synthesis occurs during erythrocyte proliferation but also the presence of some unknown GST inhibitor in the smoke.

In the case of acute exposition to contaminants, no specific studies have been done on the effects on e-GST levels. However, it can be speculated that no variation is expected if the analysis is performed within a few days from the contamination. In fact, some effect can be evident only after a few weeks given that the erythrocyte life is about 120 days and e-GST is expressed only during erythropoiesis. An apparent decreased level of e-GST would be found if the contaminant is also a GST inhibitor. We underline that a few papers reported unrealistic GST present in human serum, reasonably due to analytical artifacts as demonstrated in a re-investigation study [32]. For example, other authors studying the effects of pesticides in pesticide-sprayers found in serum a GST activity corresponding to 344 U/mL in the control group which corresponds to about 3.4 mg/mL of GST P1-1 [186]. This value is 212,000 times higher than that found in healthy subjects (8–16 ng/mL).

## 5. Utility of e-GST in Veterinary Field

e-GST may be also used as a biomarker also for veterinary purposes and to check the health status of animals reared in the farms where air, water and soil pollution are becoming a global problem. Previous studies have reported that exposure of fish to pollutants (agricultural, industrial and sewage) evolved antioxidant defense systems which included enzymes such as GST, GR, SOD and CAT [187]. Many other studies have been performed describing the detoxifying role of GSTs in different animals [188,189,190,191,192].

Vodela JK et al. investigated and compared the e-GST activities of cattle, horses, pigs, goats, dogs, rabbits, rats and mice. These authors found highest e-GST activity in mouse followed by rats, dogs, cattle, pigs, goats, horses and rabbits (this species had the lowest level of e-GST) [193].

**Figure 11:**
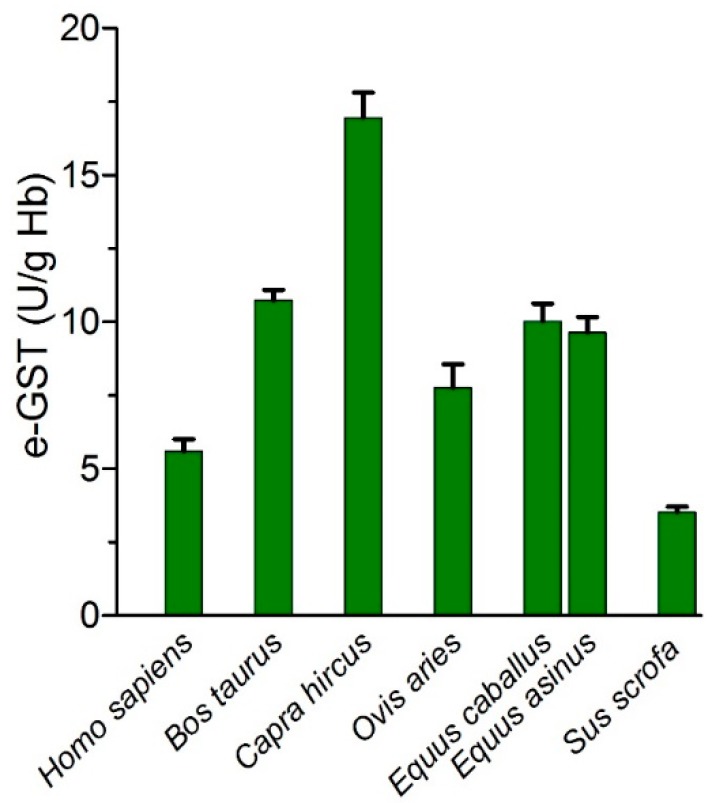
Erythrocyte glutathione transferase (e-GST) activity in some mammalian species. e-GST activity in selected mammalian species usually reared in farm and with a veterinary relevance compared to healthy human subjects (*H. sapiens*) (modified from Reference [194]).

In a different study, the possibility that e-GST may be used as an innovative and highly sensitive biomarker of blood toxicity not only for humans but also for other mammals was explored. e-GSTs from humans, *Bos taurus* (cow), *Sus scrofa* (pig), *Capra hircus* (goat), *Equus caballus* (horse), *Equus asinus* (donkey) and *Ovis aries* (sheep), all show very similar amino acid sequences, identical kinetics and stability properties (Figure 11) (Table 3) [194].

As expected, the expression of e-GST activity is species-specific; the lowest levels were found in humans and pigs, whereas the highest activity was observed in the goat. Preliminary results on cows reared in farms residing in a highly polluted area confirmed that e-GST activity is a highly sensitive parameter for detecting increased toxicity levels, as observed in humans [177]. The overexpression of e-GST in animals is a likely defense response to enhanced blood toxicity, and this behavior resembles the increased production of white blood cells in the case of a bacterial infection. An increased e-GST level in animals is, therefore, an alarm signal that must be followed up by more accurate investigations to assess the chemical nature of the contaminants.

Türkan et al. [195] studied the toxicological impact of some avermectins on human e-GST. Antiparasitic drugs, including avermectins, are used around the world in the treatment and prevention of parasitic diseases in animals, especially for animals fed externally [196]. The presence of drug residues in foods and animal products poses a serious risk to public health. The authors investigated the inhibitory effects of these toxic compounds (abamectin, doramectin, eprinomectin, ivermectin, and moxidectin), accumulated in human blood through meat, fruit, and vegetable products, on e-GST, testing them at different concentrations (0.2, 0.4, 0.6, 0.8, and 1.0 mM). The avermectins significantly inhibited the GST enzyme at the millimolar level. Therefore, avermectins should be used more carefully in agriculture and livestock.

## 6. Conclusions

**Figure 12:**
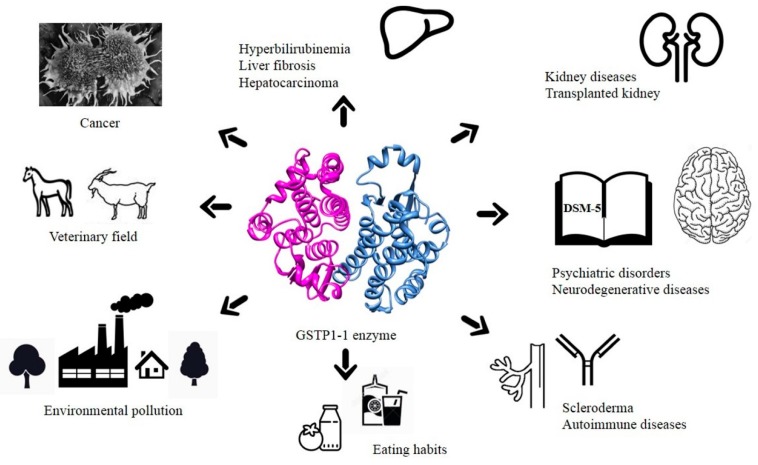
Applications of GSTP1-1 from biomedicine to environmental monitoring.

Almost all studies reported in this review support the idea that GSTP1-1 may represent a novel natural and sensible biomarker for many clinical (Table 4) and environmental applications (Figure 12). 

Nowadays, the studies about molecular biology and *GSTP1* gene polymorphisms in many different human pathologies (e.g., tumors, autoimmune, neurodegenerative and liver diseases) represent only the beginning of a conceivable use of GSTP1 as a clinical marker in biomedicine. Future researches in this field need a great sample size of the patient population examined to derive accurate statistical analyses. In cancer research, the study of structural biology, enzymology, and drug development remain extremely important mainly to inhibit the over-expressed GSTP1-1 and modulate its pro-apoptotic activity.

Furthermore, the over-expression of GSTP1-1 is a strong indication for an increase of circulating toxic compounds. Thus, it can be used to assess the gravity of kidney diseases, the efficiency of depurative procedures like the diffusive and convective dialytic techniques, and the ability of transplanted kidneys to detoxify the blood. The severity of scleroderma, possibly triggered by circulating toxins, is also correlated to the e-GST levels. However, this biosensor does not identify the chemical nature of the toxin that can be identified only through a more accurate chemical analysis. This property is reminiscent of what occurs during a bacterial infection where the increased level of lymphocytes indicates the presence of pathogens but not their identity. A useful characteristic of this biomarker is that it can be assayed in a few minutes by a very simple spectrophotometric procedure using only a few microliters of blood. The enzyme can be also stored for four/five days at 4 °C without losing its activity. Extended studies performed on a large population living in polluted areas were successfully performed giving useful indications on how to identify areas at high pollution risk. In our opinion this will be an interesting and promising field; some Italian municipalities are planning to start screening for GSTP1-1 in the near future.

## Figures and Tables

**Table 1:** Polymorphisms of GSTP1.

Allele	Alterations in Gene	Amino Acids Affected
GSTP1 * A	A313, C341, C555	Ile105, Ala114, Ser185
GSTP1 * B	G313, C341, T555	Val105, Ala114, Ser185
GSTP1 * C	G313, T341, T555	Val105, Val114, Ser185
GSTP1 * D	A313, T341	Ile105, Val114

**Table 2:** Geographic features of selected areas (modified from Reference [175]).

Selected Areas in the Frosinone District	Territorial Extension (Km^2^)	Geographic Features
Area 1	25	Nearby confluence of Sacco and Liri Rivers
Area 2	10	Close to Liri River
Area 3	90	After confluence of Sacco and Liri Rivers
Area 4	40	Near the Sacco River—Presence of industrial site
Area 5	40	Liri River flows through the area—Presence of regularized landfill and compost sites
Area 6	30	Close to an important industrial site
Area 7	60	Sacco River flows through the area
Area 8	40	Close to Sacco and Liri Rivers—Presence of incineration plant

**Table 3:** A comparison of the kinetics parameters of mammalian e-GSTs (modified from Reference [194]).

	*K*_m_		*k*_cat_ (s^−1^)
	GSH (mM)	CDNB (mM)	
*Homo sapiens*	0.11 ± 0.01	1.0 ± 0.1	79 ± 5
*Bos taurus*	0.12 ± 0.02	0.8 ± 0.2	83 ± 7
*Capra hircus*	0.14 ± 0.02	0.9 ± 0.1	85 ± 6
*Ovis aries*	0.10 ± 0.01	0.8 ± 0.2	77 ± 8
*Equus caballus*	0.10 ± 0.02	0.8 ± 0.2	82 ± 6
*Sus scrofa*	0.10 ± 0.02	0.9 ± 0.1	75 ± 7

**Table 4:** GSTP1 in diseases.

**Kidney Disease**	
Chronic Kidney Disease	[33,34,36,38,39,41,42,43,44,45,46]
Kidney Transplant	[49]
**Neurodegenerative Disease and Psychiatric Disorder**	
Parkinson’s Disease	[55,56,57,58,59]
Alzheimer’s Disease	[55,60,61,62]
Epilepsy	[64,65]
Amyotrophic lateral sclerosis	[55,67,68]
Schizophrenia	[70,73,74,75,76]
Autism	[55,78]
**Autoimmune Disease**	
Scleroderma	[84]
Others (systemic lupus erithematosus, rheumatoid arthritis, multiple sclerosis)	[86,87,88]
**Oxidative Stress**	
Oxidative Stress	[94,96,104]
**Cancer**	
Blood	[117,118,119,120,121]
Brain	[65,122,123]
Breast	[120,124,125]
Cervix	[126,128]
Ovarian	[127,128]
Endometrial	[129]
Prostate	[120,131]
Urinary bladder	[132,133,134]
Colorectal	[135,136,137]
Gastric	[115,139,140,141,142]
Esophageal	[143,144,145]
Pancreatic	[146,147]
Lung	[148,149,150]
Skin	[152,153,154]
Bone	[108,156,157,158]
**Liver Disease**	
Hepatocellular carcinoma	[159,164,165,166,167,168,169,170]
Liver fibrosis	[160,161,162]
Hyperbilirubinemia	[33]

## Abbreviations

**α-LA**	Alpha-lipoic acid
**AD**	Alzheimer’s Disease
**APC**	Antigen-presenting cells
**CAT**	Catalase
**CDAA**	choline-deficient L-amino acid-defined diet
**CDNB**	1-chloro-2,4-dinitrobenzene
**CKD**	Chronic kidney disease
**CMD**	Choline-methionine-deficient
**DEN**	Diethylnitrosamine
**DNDGIC**	dinitrosyl-diglutathionyl iron complex
**e-GFR**	Estimated-GFR
**EGF-R**	Epidermal growth factor-R
**e-GST**	Erythrocyte glutathione transferases
**ELISA**	Enzyme-linked immunosorbent assay
**ESRD**	End-stage-renal disease
**GFR**	Glomerular filtration rate
**GR**	Glutathione reductase
**GSH**	Glutathione
**GSTP**	Glutathione transferase class P
**GSTs**	Glutathione transferases
**Hb**	Hemoglobin
**HCC**	Hepatocellular carcinoma
**Hcy**	Homocysteine
**HD**	Hemodialysis
**HDF**	HemoDiaFiltration
**MHD**	Maintenance Hemodialysis
**NBDHEX**	6-(7-nitro-2,1,3-benzoxidiazol-4-ylthio) hexanol
**NFK K-DOQI**	National Kidney Foundation Kidney- Disease Outcomes Quality Initiative
**NO**	Nitric oxide
**NOS**	Nitric oxide synthase
**Nrf**	Nuclear respiratory factor
**OS**	Oxidative stress
**PD**	Parkinson’s disease
**PDB**	Protein data bank
**RBC**	Red blood cell
**ROS**	Reactive oxygen species
**SOCS-1**	Suppressor of cytokine signaling 1
**SOD**	Superoxide dismutase
**SSc**	Scleroderma or Systemic sclerosis
**T2DM**	Type 2 diabetes mellitus
**TNF-α**	Tumor necrosis factor-α
**TRAF2**	TNF-receptor factor 2
**TSGs**	Tumor suppressor genes
